# Identity and norms: the role of group membership in medical student wellbeing

**DOI:** 10.1007/s40037-013-0102-z

**Published:** 2013-12-18

**Authors:** Kathleen G. McNeill, Annelise Kerr, Kenneth I. Mavor

**Affiliations:** 1 Research School of Psychology, Australian National University, Canberra, ACT Australia; 2 ANU Medical School, Australian National University, Canberra, ACT Australia; 3 School of Psychology and Neuroscience, University of St Andrews, St Mary’s Quad, South Street, St Andrews, KY16 9JP Scotland, UK

**Keywords:** Medical students, Wellbeing, Social identity, Norms

## Abstract

Medical students experience high levels of mental health problems, which can lead to poor academic performance, substance abuse and burnout. The current paper draws on social psychology to explore the factors underpinning wellbeing in medical students. From the literature it is evident that there is a strong association between group membership and wellbeing. There is also evidence, however, that when the norms of a group prescribe unhealthy behaviours, group members who identify strongly with the group are likely to engage in those behaviours. It was hypothesized that (a) there would be a positive relationship between identification and wellbeing in medical students, (b) perceptions of norms would be positively related to unhealthy behaviour which would be negatively related to wellbeing and (c) identification would be positively related to levels of norm-related unhealthy behaviour. Ninety-two Australian medical students completed measures of identification, endorsement of norms, own behaviour in relation to norms and three indicators of wellbeing. The results supported the first hypothesis and showed only partial support for the second, suggesting a primarily positive role of group processes in medical student wellbeing. The implications for interventions to improve wellbeing in medical schools and directions for future research are discussed.

## Introduction

Medical students are known to be at high risk for mental health problems [1]. Levels of depression, anxiety and other measures of psychological distress in medical students are higher than in the general population [2]. On a personal level this distress has been found to lead to relationship problems, substance abuse and suicide [3], while on a professional level it has been linked to a reduction in academic performance [4] and may lead to cynicism and reductions in quality of patient care [3]. Much research has sought to understand the sources of distress in medical students. Financial worries, time constraints, academic stress, exams, exposure to death and human suffering and personal life events are all understood to contribute to the experience of stress and distress in medical students [3].

Whilst research has identified the many sources of stress that exist in both the personal and academic realms of functioning for medical students, less is known about the psychological processes involved in protecting some medical students from these stressors. Accordingly, there has been a call for more research which develops an understanding of the factors underpinning wellbeing in medical students [5]. It has recently been suggested that social psychological principles may provide some insight into the study of medical student wellbeing [6]. The current paper, therefore, aims to utilise the social psychological concepts of identity and norms to explore factors influencing the wellbeing of medical students.

Some researchers in the area of medical education have begun to explore the value of utilising social psychological principles. For instance, Burford [7] described the ways in which social identity theory [8] may be applied to the medical education setting to better understand the processes involved in medical students’ developing professional identities. It has also been argued that students’ development and integration of their professional identities is a fundamental part of their medical education [9]. The current research aims to extend the use of social identity principles to understanding the relationship between identity and wellbeing in medical students.

Recent research arising from the social identity approach has begun to explore the importance of group identities in maintaining both physical and mental wellbeing. For instance, it has been found that strong group memberships can improve stroke patients’ prognosis during recovery [10] and improve happiness in aged care facilities [11]. In research related more closely to the current topic, one study showed that group identities are important in easing the transition into a new programme of study [12]. Overall, research from the social psychology field is building an increasingly compelling argument that group memberships are important to wellbeing [13]. This research would suggest that medical students would benefit from a strong sense of connection (or *identification*) with their group.

When individuals *identify* with a group they see themselves as similar to other members, value their membership and behave in ways that are consistent with the group values, goals and norms [8, 14]. This means that individuals who identify more strongly with their group are more likely to engage in behaviours that are normative of the group [15]. Indeed, multiple theories in social psychology agree that individuals are most influenced by people they perceive as similar to themselves, such as fellow group members [16, 17]. This could be detrimental to the wellbeing of such individuals when the norms of the group prescribe unhealthy behaviours. Indeed, group members who identify highly with their group have been observed to make unhealthy eating choices [18] and report greater intentions to binge drink [19] when they perceive these behaviours to be consistent with group norms.

Medical education research has developed some insights into the health-related norms of medical students. For instance, it was found that medical students perceive there to be a norm of viewing mental illness as a form of weakness and as a result, students report a reluctance to seek help themselves [20]. Medical students also report high levels of binge drinking and use of other drugs and perceive this to be normative for their group [21]. In addition, it has been proposed that stress itself is seen to be a norm for the medical field [22]. Overall, it is clear that there are norms associated with medical students that are threatening to students’ health and wellbeing. Therefore, medical students who identify most strongly with their group are at most risk of behaving in ways that are consistent with these norms and thus doing most damage to their wellbeing.

**Fig. 1:**
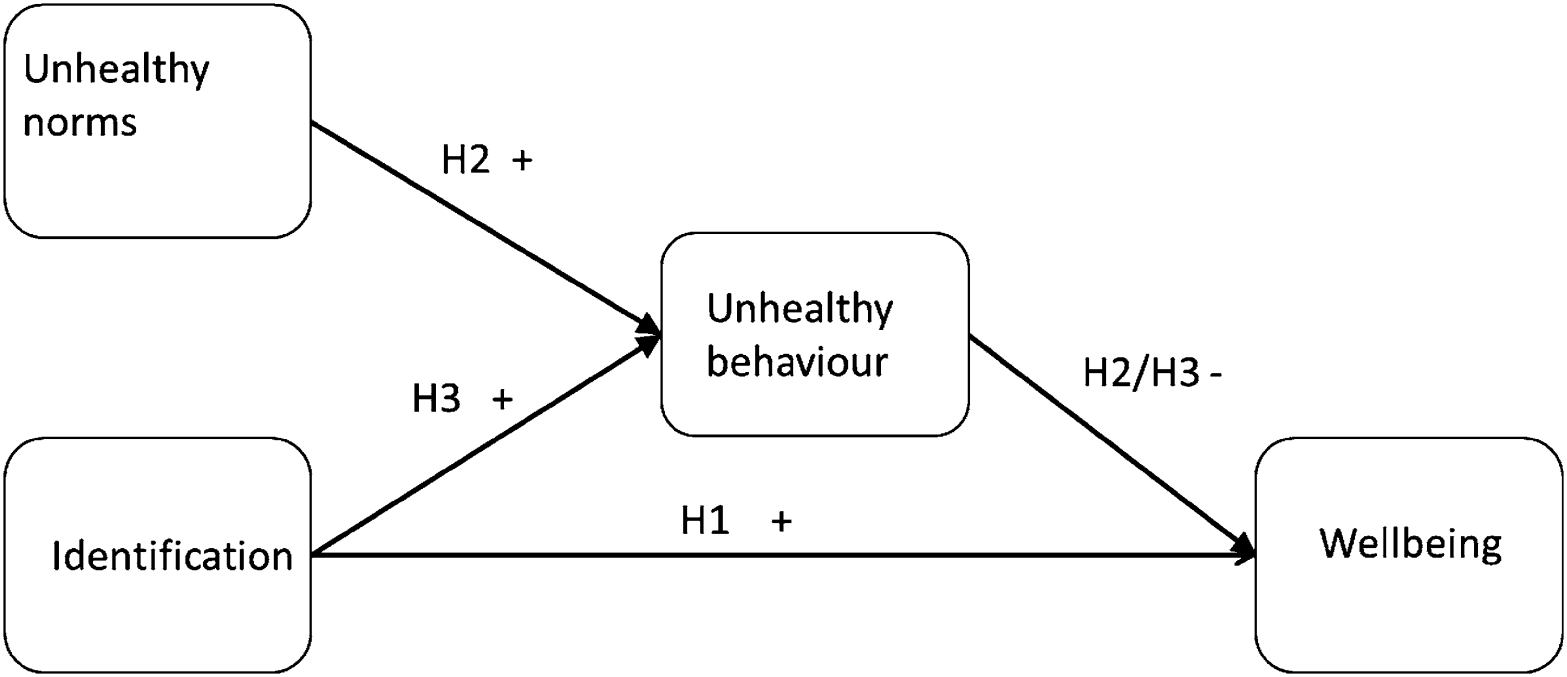
The hypothesized direct and indirect effects of identification on wellbeing

In reviewing the relevant social psychological research we have arrived at two competing hypotheses regarding medical student wellbeing. Some research suggests that group membership promotes wellbeing [13], while other research suggests that membership in groups with unhealthy norms can lead to unhealthy behaviours which reduce wellbeing [19]. Therefore, it is unclear whether group membership is beneficial or detrimental to the wellbeing of medical students. The current study will examine these two competing pathways through which identification might influence wellbeing in medical students. We explore identification with the medical student group, perceptions of group norms, health behaviours relating to those norms and measures of wellbeing to provide some insight into the psychological factors behind wellbeing in medical students. The hypotheses are outlined below and displayed in Fig. 1.

### **H1**

Medical students’ level of identification with their group will be directly and positively related to wellbeing, with those who identify most strongly with the group experiencing the greatest wellbeing.

### **H2**

Medical students’ perceptions of (negative) group norms will influence their behaviour and hence their wellbeing. Specifically, we expect to see a positive relationship between the perceived norms and reports of own norm-related behaviour, and a negative relationship between that behaviour and wellbeing.

### **H3**

Medical students’ level of identification with their group will be associated with unhealthy behaviours that are seen as normative for the group and as a result lead to reduced wellbeing.

## Method

### Participants

Ninety-two Australian medical students participated in this study. Participants were aged between 21 and 41 (*M* = 24.9, *SD* = 3.6) and 59.8 % were female (40.2 % male). Most participants were studying at the Australian National University (64.1 %), and the remainder were studying at Deakin University (10.9 %) and the University of Queensland (21.7 %). Two participants did not report their university but are likely to have been from one of the three listed. The participants were all studying medicine and ranged from those in their first year of the programme (15.2 %) to those in their fourth year (14.1 %), with the most common being second year (46.7 %), followed by third year (23.9 %). The majority of participants were born in Australia (75 %), with the remainder being born in Asia (13 %), Europe (3.2 %), United Kingdom (2 %), Africa, North America, New Zealand and Fiji (one participant from each).

### Procedure

Participants were recruited via their university online billboards and emails. The study was advertised as research investigating medical student wellbeing and participants were informed that it would take approximately 15 min to complete. Participants were prompted to follow a web link to an anonymous online survey, which included measures of identity and wellbeing.

### Measures

All measures were combined as an average of their constituent items (with negatively worded items reversed).

*Identification* with the medical student group was measured using five items (α = .78) to which participants responded on a seven-point scale with anchors ranging from ‘strongly disagree’ to ‘strongly agree’. Each of the five items corresponded to one of the five components of identification from Leach and colleagues’ [14] identification scale.

*Norms* were assessed with two items measured on a five-point scale from ‘very untrue’ to ‘very true’. The items asked participants to rate the extent that the following statements are true of medical students: ‘[Medical students] Party hard when they can to blow off a lot of steam’ (*Partying norm*) and ‘[Medical students] Don’t speak up if they are struggling with stress or other mental health problems’ (*Reluctance to seek help norm*). These two items assessed the extent to which medical students perceived these norms to be true of their medical student group. The partying norm was called this (rather than binge drinking norm) to remain more encapsulating of different behaviours (drinking and use of illicit substances) and also to avoid any ethical problems with asking (potentially underage) participants to explicitly report on illicit behaviour.

Following Terry and Hogg [15] *health behaviours* were assessed with two items that paralleled the norm items. These were placed some distance from the norm items in order to avoid simple anchoring of responses. Participants were asked to indicate how true (on a five-point scale) the following two statements were of themselves: ‘I party hard when I can to blow off steam’ (*Own partying behaviour*) and ‘I probably wouldn’t speak up if I was struggling with stress or other mental health problems’ (*Own reluctance to seek help*).

*Depression* was measured using the Centre for Epidemiological Studies Depression Scale (CES-D Scale), an instrument originally designed for use in epidemiological studies of depression in the general population [23]. The CES-D requires participants to report on the experiences of 20 depressive symptoms over the past week (α = .94), for example ‘I felt that I was not as good as other people’. Participants responded to each item using following scale: [1] rarely or none of the time (less than 1 day), [2] some or a little of the time (1–2 days), [3] occasionally or a moderate amount of the time (3–4 days), or [4] most or all of the time (5–7 days). It is important to note that this is not a diagnostic tool, and only indicates the relative presence of depressive symptoms in participants.

*Satisfaction with life* [24] was assessed using a five-item scale (α = .88). Items included ‘I am satisfied with my life’ and ‘So far I have gotten the important things I want in life’. Participants responded on a seven-point scale from ‘strongly disagree’ to ‘strongly agree’. This has previously been used as an indicator of wellbeing in student samples [12].

*Positive affect* was measured using four items (α = .88) that asked participants to rate, on a seven-point scale, how descriptive four words were of their feelings at the present moment (happy, excited, enthusiastic, proud). The first two of these items have been used to capture wellbeing in other student samples [12] and the last two items were incorporated from the Positive And Negative Affect Scale (PANAS) [25].

Depression (reversed), satisfaction with life and positive affect were standardized and averaged to form an aggregate measure of *wellbeing* with good reliability (α = .82).

## Results

### Missing data

There was 2.04 % missing data which were replaced using the Hot Deck method [26]. Using this method, the missing data are replaced by a response from another case within the same ‘deck’, which in this sample was defined by the university participants attended, their cohort year, and their gender. Myers [26] recommends this method as far superior to list-wise deletion, and as comparable to other procedures for handling missing cases.

### Descriptive statistics

The descriptive statistics are displayed in Table 1.

**Table 1:** Descriptive statistics

	Minimum	Maximum	Mean	Standard deviation
Identification	2.6	7	5.35	0.93
Partying norm	1	5	4.07	0.88
Reluctance to seek help norm	1	5	3.53	1.05
Own partying behaviour	1	5	3.01	1.36
Own reluctance to seek help	1	5	3.16	1.23
Depression	1	4	1.68	0.59
Satisfaction with life	1	7	4.63	1.38
Positive affect	1	4.75	2.60	0.96
Wellbeing	−2.26	1.56	0.01	0.86

### Correlations

Table 2 displays the correlations between the variables of interest. Strong positive correlations were observed between identification and all measures of wellbeing.

**Table 2:** Correlations

	1	2	3	4	5	6	7	8
1. Identification								
2. Partying norm	.085							
3. Reluctance to seek help norm	−.181	.105						
4. Own partying behaviour	.337**	.528**	.050					
5. Own reluctance to seek help	−.216*	−.030	.424**	.071				
6. Depression	−.419**	.040	.247*	−.093	.237*			
7. Satisfaction with life	.406**	.009	−.206*	.160	−.221*	−.566**		
8. Positive affect	.422**	.099	−.159	.194^	−.304**	−.543**	.625**	
9. Wellbeing	.491**	.027	−.241*	.175^	−.300**	−.829**	.861**	.853**

* p < .05, ** *p* < .01, ^* p* < .10

### Testing the model

**Fig. 2:**
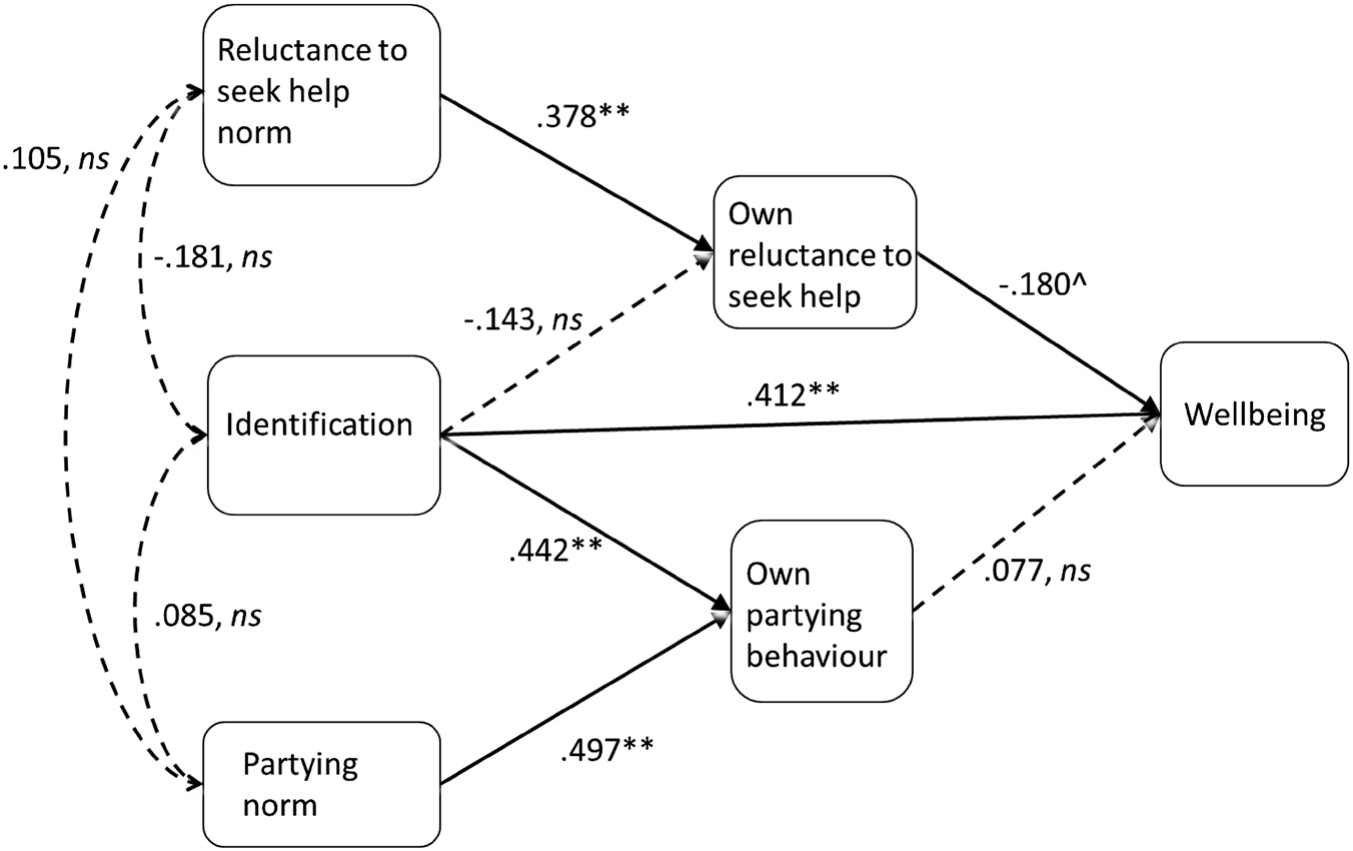
The effect of identification on wellbeing and the indirect effect of the reluctance to seek help norm on wellbeing. *p < .05, **p < .01, ^*p* < 09, n.s. p > *.09*. Standardized beta values are displayed for all *single-headed arrows* and correlation coefficients are displayed for *double-headed arrows*. Unstandardized values are shown in Table 2

The model shown in Fig. 2 was tested using Hayes’ [27] Process macro, which conducts regressions to assess the model and bootstrapping to estimate the size of the direct and indirect effects. The results of this analysis are summarised in Table 3.

**Table 3:** Summary of output from Hayes’ [24] Process macro

Outcome: own partying behaviour				
Model summary	*R* .606	*R* ^2^ .367	*F*(*df1*,* df2*) 17.02(3,88)	*p* < .001
Model	Coeff	SE	*t*	
Constant	−2.719	.942	−2.886	*p* = .005
Identification	.442	.126	3.505	*p* < .001
Partying norm	.769	.133	5.791	*p* < .001
Reluctance to seek help norm	.068	.112	.606	*p* = .546

Outcome: own reluctance to seek help				
Model summary	*R* .428	R^2^ .183	*F*(*df1*,*df2*) 6.56(3,88)	*p* < .001
Model	Coeff	SE	*t*	
Constant	2.942	.975	3.018	*p* = .003
Identification	−.189	.132	−1.449	*p* = .151
Partying norm	−.082	.137	−0.593	*p* = .554
Reluctance to seek help norm	.443	.116	3.827	*p* < .001

Outcome: wellbeing				
Model summary	*R* .540	*R* ^2^ .291	*F*(*df1*,*df2*) 7.07(5,86)	*p* < .001
Model	Coeff	SE	*t*	
Constant	−1.320	.942	−2.886	*p* = .005
Identification	.376	.092	4.071	*p* < .001
Own partying behaviour	.049	.073	.668	*p* = .506
Own reluctance to seek help	−.124	.070	−1.765	*p* = .081
Partying norm	−.043	.106	−.409	*p* = .683
Reluctance to seek help norm	−.075	.081	−.933	*p* = .354

### Direct and indirect effects

In support of hypothesis one, the direct effect of identification on wellbeing was significant (*effect* = .376, 95 % CI [.192, .559] *SE* = .092, *t* = 4.07, *p* < .001). There were no significant direct effects of either of the norm items on wellbeing. In support of hypothesis two, the indirect effect of the reluctance to seek help norm on wellbeing through own reluctance to seek help was significant (*effect* = −.058, 95 % CI [−.140, −.002] *SE* = .036). This indirect effect, however, was not significant for the partying norm; although endorsement of the partying norm had a positive association with self-reported partying behaviour, this behaviour did not have a significant impact on wellbeing. The indirect effects of identification on wellbeing through health-related behaviours were not significant, providing no support for hypothesis three in this sample.

## Discussion

This study was conducted to examine the impact of the medical student identity on wellbeing in medical students. The results were consistent with the hypothesis that group membership is beneficial to medical students, showing a strong positive relationship between identification as a medical student and measures of wellbeing. The results did not support the hypothesis that identification with the medical student group would increase unhealthy behaviours relating to group norms and therefore reduce wellbeing. However, the extent to which participants perceived those unhealthy behaviours as being normative for the group had a strong impact on their own behaviours (for both the partying and reluctance to seek help norm). In the case of the reluctance to seek help norm, this had a significant negative impact on wellbeing through reducing the likelihood that participants would ask for help if they were suffering from stress or mental health problems. Thus, results show that group membership has positive, and also potentially negative, effects on wellbeing in medical students.

The results were less clear in the case of the partying norm, where the behaviour was not seen to have an impact on wellbeing. This may be due to the ambiguity of the item used and may also represent mixed outcomes from attending parties, in that there may be both positive and negative impacts on wellbeing. Specifically, the results showed a marginally significant positive correlation between participants’ partying behaviour and positive affect. This may be related to the benefits gained from interacting with peers, such as accessing social support [28]. However, we also know from the literature that there are high levels of binge drinking at medical student parties [21], which is certainly detrimental to wellbeing. Future studies could disentangle these elements more specifically, for example by asking about drinking choices as well as controlling for social support.

It is important to consider the underlying processes that lead to the positive effects of social identification. Research from the social identity perspective has demonstrated that there are many benefits to being a group member, from making one more likely to receive help from fellow group members [29] and being more likely to be trusted by group members [30] to being more likely to have fellow group members laugh with you [31] and follow your lead [32]. Group membership also provides a platform for individuals to receive and benefit from social support [28]. It is likely that the highly identifying medical students in the current sample were enjoying some of these benefits of their group membership. Further research could examine more mediators of the identification and wellbeing relationship, by measuring some of the above factors.

The directionality of the relationship between identification and wellbeing should be considered. Given the correlational nature of the study, it is not clear whether, as suggested, identification improves wellbeing, or in fact higher levels of wellbeing improve identification. We suggest that this relationship is likely to be a bidirectional one, with both identification and wellbeing influencing each other. As we have discussed, there are many benefits to identification with a group, many of which facilitate wellbeing. We also know that symptoms of poor wellbeing (such as depression) include social withdrawal, which makes participation in a group very difficult. Importantly, this bidirectional relationship suggests that changes in either identification or wellbeing are likely to result in changes in the other.

Many studies in the field of social psychology have demonstrated that both identification and perceived norms can be influenced and changed [18]. Thus, on a practical level, it may be possible to create interventions in medical schools that increase the benefits of group membership and decrease the costs. Medical schools often facilitate group identification of their students by setting up activities in which students cooperate to work towards a common goal. However, the competitive nature of medical school may undermine a shared group identity by encouraging students to compete with each other, rather than cooperate. Strategies such as utilising a pass/fail, rather than letter grading system may reduce this competitive atmosphere [33].

It may also be possible to design interventions that target the perceptions of health-related norms in medical school. It has been shown that group members’ perceptions of norms can be changed by messages delivered by in-group members and that this change in perception of norms can result in changes in health behaviours [18]. Thus student representatives may be able to influence their fellow students by sharing positive health messages, for example by encouraging students to speak up if they are struggling with stress and by talking about ways to enjoy parties without binge-drinking.

The findings of the current study allow us to understand more about the factors that may contribute to vulnerability in some medical students. The results suggest that students with low levels of identification with their group would be at higher risk for mental health problems. There may be some students for whom it is difficult to create or maintain a sense of connection to their student group. For instance, students who undertake rural placements are physically removed from the company of their peers, sometimes being the only medical student in a hospital. Under these conditions it may be difficult to maintain a strong medical student identity. Ongoing interactions and cooperative activities between rural and non-rural students may be a way to facilitate the maintenance of a group identity. There may be other conditions under which the medical student identity may be difficult to maintain. For example, when one’s perception of what ‘medical students can do’ does not fit with perceptions of one’s own skills [7]. The current results suggest that these threats to students’ identities are also threats to their wellbeing. Future research should investigate the conditions under which the medical student identity is difficult for students to access or maintain and how this impacts upon their wellbeing.

## Conclusion

This study introduces concepts from the social psychological field to the study of wellbeing in medical students. It demonstrates the value of exploring elements of group membership and its relationship to wellbeing. The results demonstrate that there is a positive relationship between social identification and medical student wellbeing, suggesting that activities that promote cooperation and shared experiences amongst medical students will be helpful for their wellbeing. The findings also suggest that some norms associated with the medical student identity may be detrimental to wellbeing. Directions for future research in this area include further exploration of the mediators and moderators of the identity-wellbeing relationship and on a more applied level, investigation of interventions that shift perceptions of norms and boost identification for at-risk students to improve their wellbeing.

## Essentials

- Medical student wellbeing is positively associated with students’ identification with their medical student group.
- Some unhealthy norms associated with the medical student group can lead to unhealthy choices, which may be detrimental to students’ wellbeing.
- Group membership has positive and also potentially negative impacts on medical students’ wellbeing.
- Social psychological principles make a valuable contribution to understanding wellbeing in medical students.
